# KIF2A decreases IL-33 production and attenuates allergic asthmatic inflammation

**DOI:** 10.1186/s13223-022-00697-9

**Published:** 2022-06-19

**Authors:** Zhengxia Wang, Jingjing Wu, Jingxian Jiang, Qiyun Ma, Meijuan Song, Tingting Xu, Yanan Liu, Zhongqi Chen, Yanmin Bao, Mao Huang, Mingshun Zhang, Ningfei Ji

**Affiliations:** 1 Department of Respiratory and Critical Care Medicine, The First Affiliated Hospital of Nanjing Medical University, Nanjing, China; 2 Jiangsu Province Engineering Research Center of Antibody Drug, NHC Key Laboratory of Antibody Technique, Department of Immunology, Nanjing Medical University, Nanjing, China; 3 Department of Respiratory Medicine, Shenzhen Children’s Hospital, Shenzhen, China

**Keywords:** Asthma, IL-33, KIF2A, Autophagy, Epithelial, mTORC1

## Abstract

**Background:**

The microtubule-dependent molecular motor protein Kinesin Family Member 2A (KIF2A) is down-regulated in asthmatic human airway epithelium. However, little is known about the roles of KIF2A as well as the possible underlying mechanisms in asthma.

**Methods:**

House dust mite (HDM) extract was administered to establish a murine model of asthma. The expression of KIF2A, IL-33 and the autophagy pathways were detected. The plasmid pCMV-KIF2A was used to overexpress KIF2A in the airway epithelial cells in vitro and in vivo. IL-4, IL-5, IL-33 and other cytokines in bronchoalveolar lavage fluid (BALF) and lung tissues homogenates were measured.

**Results:**

In response to the challenge of house dust mite (HDM) in vitro and in vivo, airway epithelial cells displayed decreased production of KIF2A. Meanwhile, autophagy and IL-33 were increased in HMD-treated epithelial cells. Mechanistically, KIF2A decreased autophagy via suppressing mTORC1 pathway in HDM-treated epithelial cells, which contributed to the reduced production of IL-33. Moreover, in vivo KIF2A transfection reduced IL-33 and autophagy in the lung, leading to the attenuation of allergic asthma.

**Conclusion:**

KIF2A suppressed mTORC1-mediated autophagy and decreased the production of epithelial-derived cytokine IL-33 in allergic airway inflammation. These data indicate that KIF2A may be a novel target in allergic asthma.

**Supplementary Information:**

The online version contains supplementary material available at 10.1186/s13223-022-00697-9.

## Background

Allergic airway inflammation (AAI) orchestrates the initiation and progression of allergic asthma [1], which has afflicted almost 400 million people as of 2020 [2]. The pulmonary epithelium plays a central role in directing and propagating AAI [3]. Upon stimulation by house dust mites (HDM) and other allergens, epithelial cells release alarmins, i.e., IL-25, IL-33, and thymic stromal lymphopoietin (TSLP) [4]. IL-33 is a tissue-derived nuclear cytokine from the IL-1 family [5]. IL-33 applied directly to the airways is sufficient to induce AAI, which includes eosinophil infiltration and Th2-type responses, while blocking IL-33 significantly abrogates these changes [6]. Endogenous IL-33 is released from the nucleus in its full-length form (IL-33_FL_) after cellular damage or necrotic cell death [7]. The mature forms of IL-33 that are generated by inflammatory proteases have ~ 30-fold higher biological activity than the IL-33_FL_ precursor [8]. A previous study showed that the cleavage and activation of IL-33_FL_ by allergen proteases play a crucial role in the rapid induction of allergic airway inflammation following exposure to allergens [9].

Autophagy is a tightly orchestrated process that sequesters misfolded proteins, damaged or aged organelles, and mutated proteins in double membrane vesicles called autophagosomes that ultimately fuse with lysosomes, leading to the degradation of the sequestered components [10]. Autophagy plays a role in asthma pathogenesis; autophagy markers are increased in patients with asthma [11]. In a similar manner, inhalation of ovalbumin increased autophagy in airway tissues in a mouse model [12]. Intranasal administration of IL-33 was shown to promote IL-13-dependent autophagy and thereafter regulate mucus secretion by airway epithelial cells [13]. However, it remains to be explored whether autophagy regulates IL-33 expression in allergic airway inflammation.

The kinesin superfamily proteins (KIFs) are a conserved class of microtubule-dependent molecular motor proteins exhibiting adenosine triphosphatase activity, and are important in mitosis, meiosis, and macromolecular transport [14]. KIF2A belongs to the kinesin-13 family, and regulates microtubule (MT) end dynamics through its ATP-dependent MT-depolymerase activity [15]. RNA-seq showed that KIF2A was downregulated in differentiated asthmatic human airway epithelial cultures [16]. Knockdown of KIF2A, which increased lysosomal perinuclear localization, reduced mTORC1 activity and increased autophagosome synthesis under starvation conditions [17]. However, little is known about the mechanisms by which KIF2A regulates autophagy in respiratory epithelial cells and AAI. We hypothesized that KIF2A may regulate mTOR and autophagy, thereby regulating IL-33 in AAI. To test this hypothesis, we assayed KIF2A and autophagy proteins and assessed their effects on IL-33 production in airway epithelial cells challenged with HDM in vitro and in vivo.

## Materials and methods

### Animals

Specific pathogen-free female C57BL/6 J mice aged 6 to 8 weeks were obtained from Nanjing Medical University (Nanjing, China). All mouse experiments and tissue sample collections were carried out in accordance with the guidelines and procedures approved by the Institutional Animal Care and Use Committee of Nanjing Medical University (IRB: 1709011).

### Cell lines and cell culture and treatment

The human bronchial epithelial cells lines 16-HBE, mouse pulmonary epithelial cell lines MLE-12 and alveolar epithelial type 2 (AT2) were cultured in DMEM/F12 (Gibco, Massachusetts, USA) supplemented with 10% fetal bovine serum (HyClone, Logan, USA), 100 μg/mL penicillin, and 100 μg/mL streptomycin (60162ES76, Yeasen, Shanghai, China). All incubations were carried out in 5% CO2 in air at 37 °C. For the experiments, cells were seeded and grown to 80–90% confluency in 24-well culture plates (3524, Corning, New York, USA) and exposed to whole-body HDM extract from *Dermatophagoides pteronyssinus* (Greer Laboratories, Lenoir, USA) for the indicated durations. Autophinib (HY-101920, MedChemExpress, New Jersey, USA) was used as an autophagy inhibitor in the study. Usnic acid (HY-N0656A, MedChemExpress, New Jersey, USA) was used to inhibit the phosphorylation of mTOR downstream effectors P70S6K. Cell lysates and cell-free supernatant were collected for further analysis.

### AT2 cell isolation and culture

A crude single lung cell suspension was isolated from 6 to  8-week-old B6 mice as previously described [18]. Mice were anaesthetized by intraperitoneal injection of pentobarbital sodium (70 mg/kg), and the lung vasculature was perfused through the right ventricle with PBS. The trachea was punctured, and the lungs were injected with 1 ml digestion buffer (0.25% trypsin/2 mM EDTA (25200056, Gibco, Massachusetts, USA) and 1 mg/ml elastase (Worthington) for 5 min. Immediately following digestion, 0.5 mL of 45 °C 1% low melting agarose in Ca2^+^ Mg2^+^-free PBS was instilled into the lungs for 2 min. The lungs were removed from the thorax and digested for 45 min at room temperature on a shaker. The lobes were then separated, minced, and digested with 0.01% DNase (Sigma) for 10 min at room temperature. The resulting cell suspensions were filtered first through a 100 μm filter and then a 40 μm filter (Falcon, Cockeysville, MD).

AT2 cells were isolated using the “panning method” which purifies cells using IgG-coated plates [19]. Briefly, three 100-mm bacteriologic plastic dishes (430,167, Corning, New York, USA) were coated with 3 to 5 ml each of 0.5 mg/ml IgG (Sigma), and IgG was allowed to adhere for 3 h at 37 °C. The cell suspension was added at a density of 20 to 30 × 10^6^ cells/10 ml medium/100-mm dish. Plates were placed in an incubator for 30 min and then removed from the incubator and carefully and gently tipped back and forth 3 times. The unattached cells were removed and centrifuged at 300*g* for 10 min. The pellet was resuspended in DMEM/F12 with 10% FBS and cultured in 24-well plates.

### Western blot

Total cellular protein or tissue was collected following lysis in RIPA lysis buffer (87,788, Thermo Fisher Scientific, Massachusetts, USA) with Protease Inhibitor Cocktail (78,430, Thermo Fisher Scientific, Massachusetts, USA) on ice and centrifugation for 10 min at 12,000 rpm at 4 °C. The supernatant was then transferred to a new tube and denatured in sodium dodecyl sulfate–polyacrylamide gel electrophoresis (SDS-PAGE) loading buffer (P0015, Beyotime Biotech, China) with heating at 100 °C for 10 min. The supernatant was then stored at − 80 °C. The proteins were separated by 12% SDS-PAGE. After electrophoresis, the separated proteins were transferred to 0.45 µm or 0.22 µm polyvinylidene difluoride membranes (Merck Millipore, USA) using a wet transfer method. Nonspecific sites were blocked with 5% nonfat milk in TBS-Tween 20 (TBST; 25 mM Tris [pH 7.5], 150 mM NaCl, and 0.1% Tween 20) for 1 h, and the blots were incubated with primary antibodies, including anti-β-actin (4970L, Cell Signaling Technology, Massachusetts, USA), anti-KIF2A (ab197988, Abcam, Cambridge, England), anti-LC3B (ab192890, Abcam, Cambridge, England), anti-ATG5 (ab108327, Abcam, Cambridge, England), anti-IL-33 (ab187060, Abcam, Cambridge, England), anti-P-4EBP1 (2855, Cell Signaling Technology, Massachusetts, USA), anti-4EBP1 (9644, Cell Signaling Technology, Massachusetts, USA), anti-p-p70 S6 Kinase (Thr389) (9234, Cell Signaling Technology, Massachusetts, USA), anti- p-p70 S6 Kinase (Ser371) (9208, Cell Signaling Technology, Massachusetts, USA), and anti-p70 S6 Kinase (2708, Cell Signaling Technology, Massachusetts, USA), overnight at 4 °C. HRP-linked anti-rabbit IgG (7074, Cell Signaling Technology, Massachusetts, USA) was used to detect antibody binding. After the membranes were treated with Immobilon Western Chemiluminescent HRP Substrate (WBKLS0500, Merck Millipore, USA), the binding of specific antibodies was visualized using a Syngene G:BOX Imaging System and was analyzed with ImageJ.

### Reverse transcription and qPCR

Total RNA was isolated from frozen tissues or cells using TRIzol (Invitrogen), and cDNA was synthesized using 5X All-In-One RT MasterMix (G490, Abm, Zhenjiangg China) according to the manufacturer’s instructions as described previously [20]. qPCR analysis was performed using a StepOnePlus Real-Time PCR System (ABI, USA) in conjunction with SYBR Advantage qPCR Premix (63976, TaKaRa Bio). The cycling conditions were 95 °C for 30 s, followed by 95 °C for 5 s and 60 °C for 30 s for up to 40 cycles and dissociation at 95 °C for 5 s, 60 °C for 30 s, and a final extension at 95 °C for 15 s. The relative abundance of gene targets was determined by the comparative CT (cycle threshold) number normalized against the tested β-actin comparative CT. The primers used are shown in Table 1.

**Table 1:** Primers in the study

Name	Sequence
β-actin-F	GAGAAGCTGTGCTATGTTGCT
β-actin-R	CTCCAGGGAGGAAGAGGATG
KIF2A-F	ATTTTCTCTCATTGACCTGGCTG
KIF2A-R	ACTCCTTGAGTGCTAAAAGGC
IL-33-F	GGAGGACCAGCTAGGGGGAG
IL-33-R	GGGCTGATCTGAGGGTTGCC
IL-25-F	ACAGGGACTTGAATCGGGTC
IL-25-R	TGGTAAAGTGGGACGGAGTTG
TSLP-F	AGTCCTCGATTTGCTCGAACT
TSLP-R	AGTCCTCGATTTGCTCGAACT

### HDM sensitization/challenge protocol

C57BL/6 J mice were randomly divided into 4 groups: control, HDM, HDM treated with an empty vector (Vehicle) (PS100001, OriGene Tech, Rockville, MD, USA), and HDM treated with pCMV6-KIF2A (MR210157, OriGene Tech). Mice in the latter 3 groups were exposed intratracheally to HDM according to the established 14-day model [21]. The mice anesthetized with isoflurane received 100 μg HDM in 40 μl of normal saline (NS) on Day 0 and 10 μg HDM in 40 μl of normal saline (NS) on Days 7–11 intratracheally to induce allergic lung inflammation. Mice exposed to 40 μl NS according to the HDM protocol served as controls. To explore whether KIF2A was involved in allergic airway inflammation pathogenesis, 50 µg pCMV6-KIF2A-ORF or vehicle was complexed with in vivo-jetPEI™ (201-50 g, Polyplus Transfection, New York, USA) in 200 µl of 5% glucose solution and injected into the tail vein before the HDM challenge on days 6 and 10 [22]. The mice were sacrificed 3 days after the final challenge, and bronchoalveolar lavage fluid (BALF) and lung tissues were collected for analyses.

### Measurement and analysis of airway responsiveness

Mice were anesthetized with 70 mg/kg pentobarbital and 1.8 g/kg urethane followed by 0.5 mg/kg pancuronium bromide, and the mice were tracheotomized 72 h after the final challenge [23]. Airway hyperreactivity (AHR) was measured in response to increasing doses of acetylcholine via a flexiVent FX system with an integrated FX1 module (Scireq, Montreal, QC, Canada) under general anesthesia as described previously [24].

### Bronchoalveolar lavage fluid and serum analysis

After AHR measurement, whole blood was collected without anticoagulant and incubated for 2 h at room temperature, and serum was isolated by centrifugation at 2000*g* for 10 min. Bronchoalveolar lavages were conducted by insertion of a cannula into the trachea, which was fixed by a suture. BAL fluids were taken by slow injection and subsequent aspiration of PBS-EDTA solution (1 mM EDTA, PBS, 500 μl × 3; 85 to 90% of the lavage volume was recovered) with a 1 -mL syringe. BALF from each mouse was centrifuged at 500*g* for 10 min at 4 °C, cell pellets were resuspended in 100 µl phosphate-buffered saline (PBS), and differential cell counts were performed using standard morphological criteria after Wright-Giemsa staining. The BALF was collected, divided into equal portions, and frozen at − 80 °C until further preparation.

### Lung histology

Lungs were fixed in 4% paraformaldehyde; the left lung of each mouse was embedded in paraffin according to standard procedures. Sections (5 µm) were mounted on slides for histological or immunohistochemistry (IHC) analysis. Hematoxylin and eosin staining was used to evaluate changes in lung inflammation. Mucus secretion was assessed by PAS staining. Images were visualized with a Zeiss Axio Examiner microscope. The severity of peribronchial inflammation was graded semiquantitatively for the following features: 0, normal; 1, few cells; 2, a ring of inflammatory cells 1 cell layer deep; 3, a ring of inflammatory cells 2–4 cells deep; 4, a ring of inflammatory cells 4 cells deep. The numerical scores for the abundance of PAS-positive mucus-containing cells in each airway were determined as follows: 0, < 0.5% PAS-positive cells; 1, 5–25%; 2, 25–50%; 3, 50–75%; and 4, > 75% [20].

### ELISA

The levels of IL-4, IL-5, IFN-γ (431105, 430805, 431205, Biolegend, San Diego, CA), Eotaxin, IL-33 (DY420, DY3626, R&D Systems, Minnesota, USA) and IL-13 (96–900-K207, PeproTech, Offenbach, Germany) in lung homogenates and total IgE (555248, BD Biosciences, San Jose, CA) in sera were measured using commercial ELISA kits according to the instructions provided by the manufacturers.

### Immunohistochemistry and immunofluorescence

Formaldehyde-fixed mouse lungs were dehydrated, paraffin embedded, and sectioned (5 μm thickness). Sections were rehydrated, quenched with 3% hydrogen peroxide, incubated in citric buffer for antigen retrieval, and blocked with the avidin/biotin blocking system and then 5% normal goat serum, followed by overnight incubation at 4  C with primary anti-KIF2a antibody. Tissue sections were then incubated with horseradish peroxidase-conjugated secondary antibodies for 1 h at room temperature. The staining was visualized with 3,3′-diaminobenzidine (DAB, Vectastain, Vector Laboratories, USA) and background stained with hematoxylin. All KIF2A-stained sections were scanned with a Zeiss Axio Examiner microscope, and representative photos were chosen and presented for each stimulus group in the study.

For immunofluorescence, slides or cells were then fixed with 4% paraformaldehyde, rinsed twice with PBS, and permeabilized in PBS 0.5% Triton X-100. Samples were incubated with 5% normal goat serum for 1 h at room temperature (RT) and stained in blocking buffer with anti-LC3B antibodies (1:500 in blocking buffer), anti-KIF2A (1:100, sc-272471, Dallas, Texas, USA), or anti-Prosurfactant Protein C (1:50, ab170699, Abcam, Cambridge, England) overnight at 4 °C. After samples were washed with PBS, secondary goat anti-rabbit IgG antibodies conjugated with Alexa Fluor 647 (1:1000) or goat anti-rabbit IgG antibodies conjugated with Alexa Fluor 555 (1:1000) were added to the samples and incubated for 1 h in the dark at RT. The samples were finally mounted with DAPI (36308ES20 Yeasen, China).

### Transfection

MLE-12 cells were seeded into 24-well plates. Before transfection, 500 ng vehicle or KIF2A-ORF plasmids was mixed with 2.5 μL of Hieff TransTM Liposomal Transfection Reagent (40802ES02, Yeasen, China) in 100 μL of DMEM for 25 min. After 25 min, the plasmid-transfection reagent mixture was added to the wells (100 μL/well). The transfection medium was discarded after 6 h, and the cells were cultured in medium containing 10% FBS for no less than 24 h before further analysis.

### Statistical analysis

Results are expressed as the mean ± SEM. Comparisons among three or more groups were performed by one-way ANOVA followed by the Newman-Keuls post hoc test. Significance between two groups was assessed by unpaired or paired *t* tests. Statistical analyses were performed using GraphPad Prism 7.0

## Results

### Decreased pulmonary expression of KIF2A in a mouse model of AAI

**Fig. 1:**
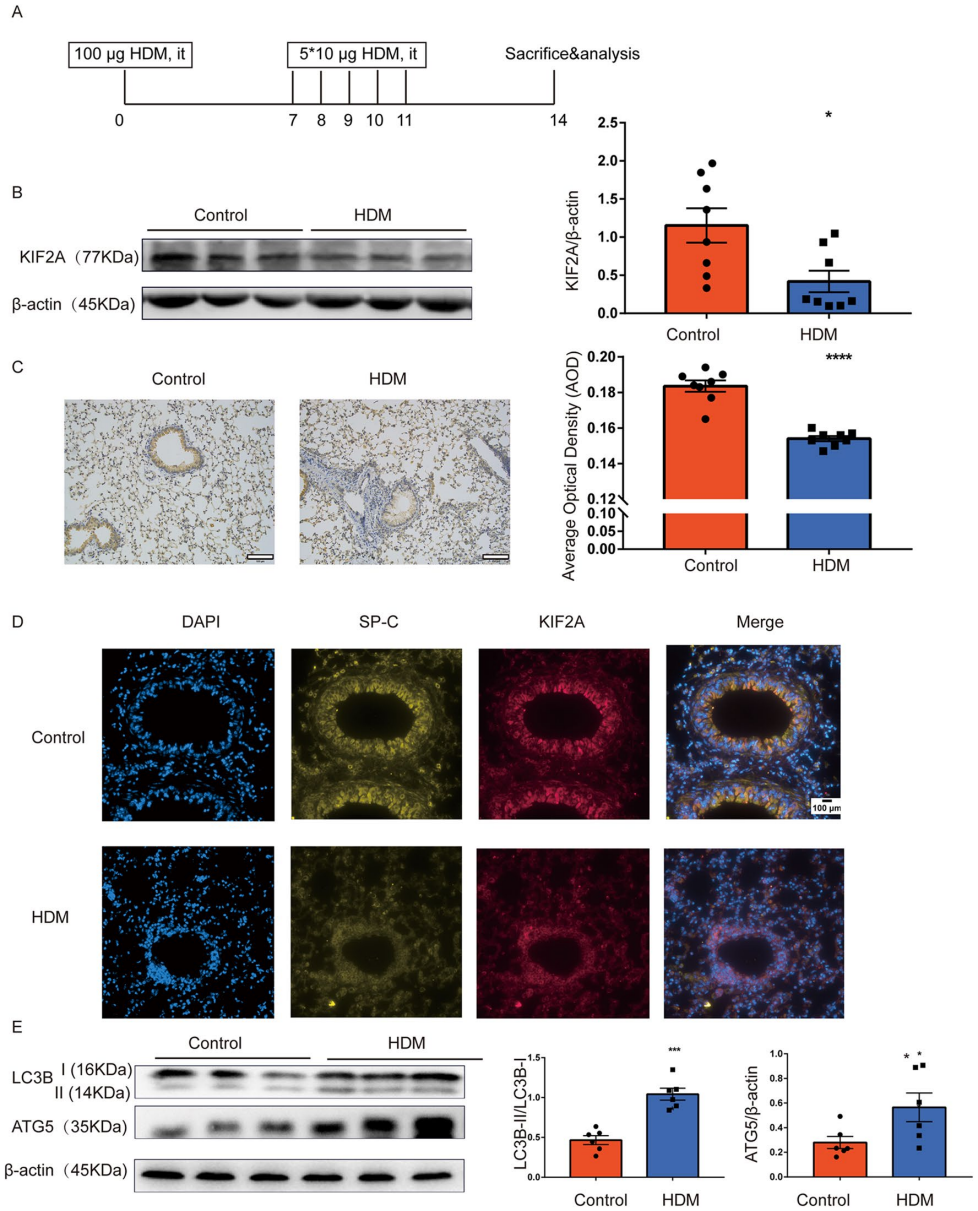
Decreased KIF2A and increased autophagy in a mouse model of allergic airway inflammation. **A** The mouse model of allergic airway inflammation. **B** KIF2A in lung tissues from mice in the control group and the HDM group was measured with Western blotting and the density quantification of KIF2A was expressed as a ratio relative to β-actin (**C**) Immunohistochemical staining for KIF2A in lung sections from mice in the control group and the HDM group. Scale bar, 100 μm and AOD was measured with imageJ. **D** Immunofluorescent staining for KIF2A and prosurfactant protein C (AT2 cell marker) in lung sections from mice in the control group and the HDM group. Scale bar, 100 μm. **E** The level of autophagy (LC3B and ATG5) in lung tissue from mice in the control group and the HDM group was measured with Western blotting and the density quantification of LC3B and ATG5 was expressed as a ratio relative to β-actin. Data are presented as mean ± SEM of independent experiments with similar results (n = 6 ~ 8). *p < 0.05, **p < 0.01, ***p < 0.001, ****p < 0.0001

In a well-established HDM-induced murine model of AAI [20] (Fig. 1A), we first assessed the expression of KIF2A. The expression of KIF2A was downregulated in the lungs of HDM-exposed mice compared to the lungs of control mice (Fig. 1B). We further confirmed this observation by immunocytochemistry, as evidenced by the decreased average optical density (Fig. 1C). Based on the immunofluorescent staining results for KIF2A and prosurfactant protein C [25], we found that KIF2A was mainly expressed on lung SP-C^+^ epithelial cells (Fig. 1D). Immunofluorescent analysis also showed the downregulation of SP-C in HDM-exposed mice, which was in accordance with a previous study [26]. Previous study showed KIF2A regulated autophagy under starvation conditions [17]. The autophagy level was also measured using Western blotting. As shown in Fig. 1E, LC3B-II/LC3B-I and ATG5 were upregulated, suggesting that HDM increased autophagy in the allergic airway inflammation model. Collectively, these results showed that HDM decreased pulmonary expression of KIF2A and upregulated autophagy in a mouse model of AAI.

### HDM exposure induced downregulation of KIF2A in epithelial cells

**Fig. 2:**
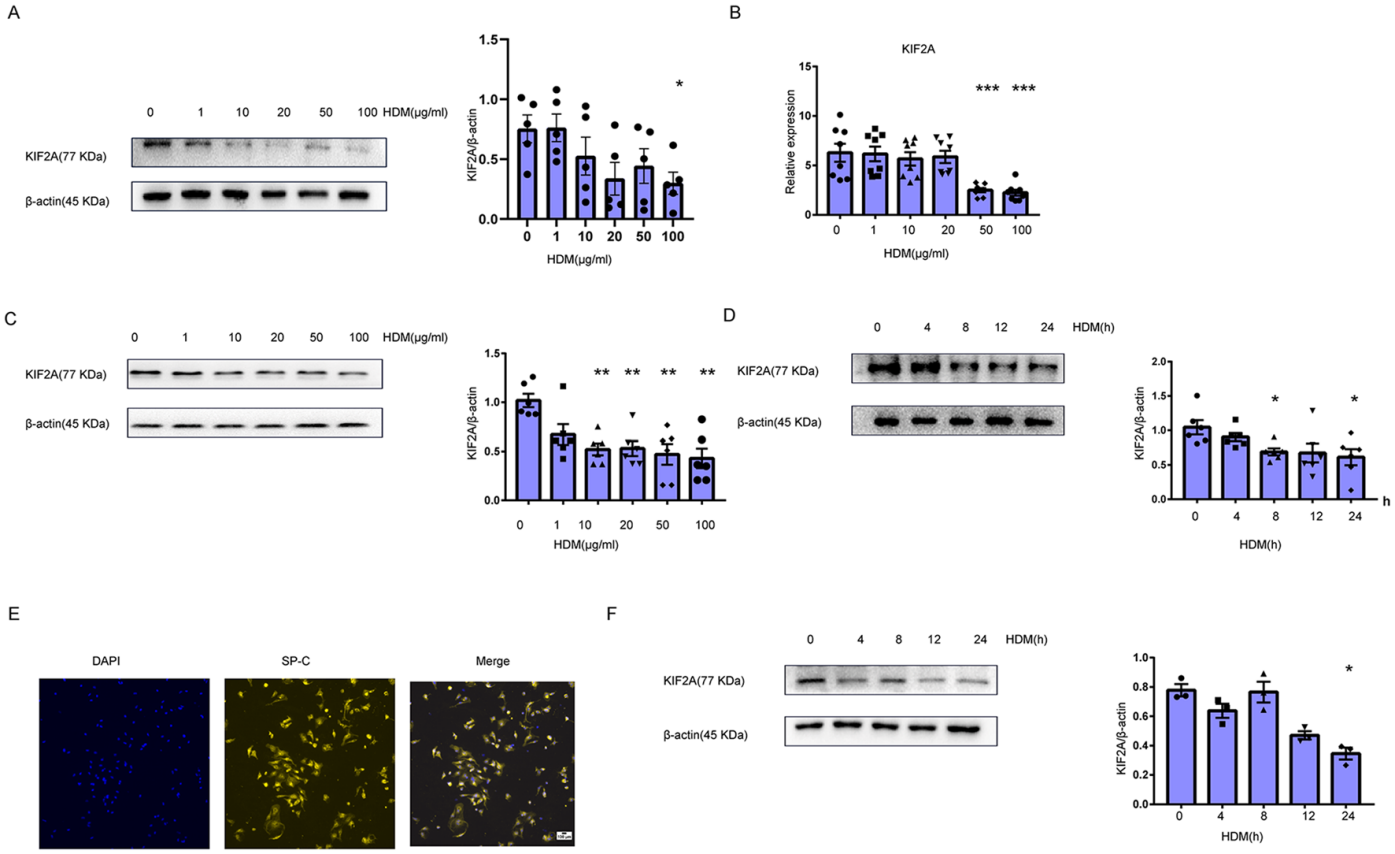
HDM decreased KIF2A expression in epithelial cells. **A** 16-HBE were left untreated or treated with different concentrations of HDM for 24 h. The expression of KIF2A was measured with Western blotting and the density quantification of KIF2A was expressed as a ratio relative to β-actin (**B**) MLE-12 cells were left untreated or treated with different concentrations of HDM for 24 hous. The expression of KIF2A was measured by qPCR. **C** MLE-12 cells were left untreated or treated with HDM as described in (**B**), and the KIF2A expression levels were examined using a Western blot assay. The density quantification of KIF2A was expressed as a ratio relative to β-actin (**D**) MLE-12 cells were left untreated or treated with HDM (50 μg/mL) for the indicated time periods; then, the KIF2A expression levels were measured with Western blotting and the density quantification of KIF2A was expressed as a ratio relative to β-actin (**E**) Immunofluorescence staining of SP-C (alveolar epithelial cell marker) in primary ATII cells. Scale bar, 100 μm. **F** Primary epithelial cells were left untreated or treated with HDM (50 μg/mL) for the indicated time periods; then, the expression of KIF2A was measured using western blotting. The density quantification of KIF2A was expressed as a ratio relative to β-actin. Data are presented as mean ± SEM of independent experiments with similar results (n = 3 ~ 8). *p < 0.05, **p < 0.01, ***p < 0.001, ****p < 0.0001

RNA-seq showed that KIF2A was downregulated in differentiated asthmatic human airway epithelial cultures [16] (Additional file 1: Fig. S1). To explore whether HDM directly affected the expression of KIF2A in epithelial cells, human bronchial epithelial cells(16-HBE) was stimulated with different doses of HDM, and then KIF2A expression was measured As shown in Fig. 2A, KIF2A expression was decreased following HDM treatment at different doses. The pulmonary epithelial cell line MLE-12 was also stimulated with HDM, and then KIF2A expression was measured by qPCR and western blotting 24 h after HDM exposure. As shown in Fig. 2B, C KIF2A expression was significantly decreased after HDM treatment at different doses, and the lowest expression of KIF2A was observed upon exposure to 50 μg/mL HDM.

Next, we determined the KIF2A expression levels in MLE-12 cells under treatment with a single dose of HDM extract (50 μg/mL) for different time periods. As shown in Fig. 2D, the time-dependent repression of KIF2A expression was readily observed in MLE-12 cells. To further confirm the above results related to KIF2A in MLE-12 cells, we examined KIF2A expression in mouse primary type II alveolar epithelial type 2 (AT2) cells under HDM treatment. The purified primary AT2 cells expressed SP-C (Fig. 2E). As shown in Fig. 2F, a significant downregulation of KIF2A expression was observed in the primary cells upon HDM exposure. Taken together, these data indicated that KIF2A was downregulated in epithelial cells in response to HDM stimulation.

### HDM induced alarmin cytokines expression in epithelial cells

**Fig. 3:**
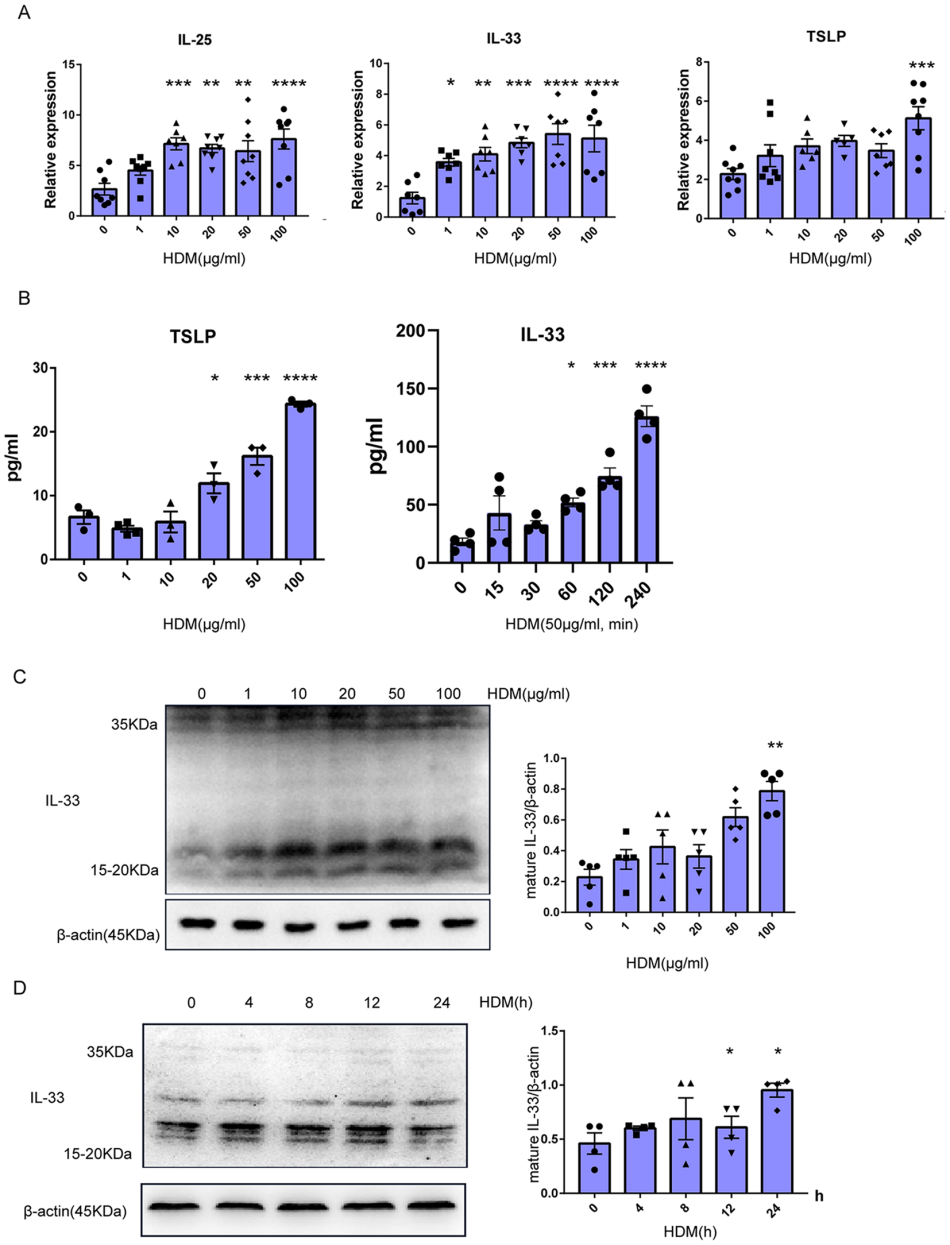
HDM induced IL-25, IL-33, and TSLP secretion in epithelial cells. **A** MLE-12 cells were left untreated or treated with different concentrations of HDM for 24 h. The expression levels of IL-25, TSLP, and IL-33 were measured using qPCR. **B** MLE-12 cells were left untreated or treated with HDM as described in (**A**), and the TSLP and IL-33 expression levels in supernatant were examined using ELISA. The level of IL-33 in supernatant was measured following HDM treatment at the indicated time. **C** MLE-12 cells were left untreated or treated with HDM as described in (**A**), and the IL-33 expression levels were examined using Western blotting. the density quantification of IL-33 was expressed as a ratio relative to β-actin (**D**) Primary epithelial cells were left untreated or treated with HDM extract (50 μg/mL) for the indicated time periods; then, the expression of IL-33 was measured using Western blotting and the density quantification of IL-33 was expressed as a ratio relative to β-actin. Data are presented as mean ± SEM of independent experiments with similar results (n = 4 ~ 8). *p < 0.05, **p < 0.01, ***p < 0.001, ****p < 0.0001

The main epithelial-derived cytokines IL-33, IL-25, and TSLP play critical roles in the genesis of Th2-type inflammation in the asthmatic airway mucosa by directly activating ILC2s and inducing Th2-type T cells differentiation [27]. To test whether alarmin cytokines could be induced by HDMs in vitro, MLE-12 cells were treated with HDMs. As shown in Fig. 3A, the alarmin cytokines IL-33, IL-25, and TSLP were upregulated following HDM treatment. In line with the qPCR results, ELISA showed that TSLP protein levels were upregulated in HDM-exposed epithelial cells supernatant (Fig. 3B). IL-33 could not be detected 24 h after HDM treatment in the supernatant. However, IL-33 was upregulated in HDM-exposed epithelial cells supernatant within several hours (Fig. 3B). Indeed, the expression of IL-33 protein, which was mainly found in its mature form rather than the full-length form, was increased in HDM-treated epithelial cells (Fig. 3C). As expected, this significant upregulation of mature IL-33 expression was readily observed in the primary cells upon HDM exposure (Fig. 3D). In summary, HDMs induced the expression of alarmin cytokines (IL-25, IL-33, and TSLP) in respiratory epithelial cells.

### Autophagy regulated IL-33 expression in HDM-treated epithelial cells

**Fig. 4:**
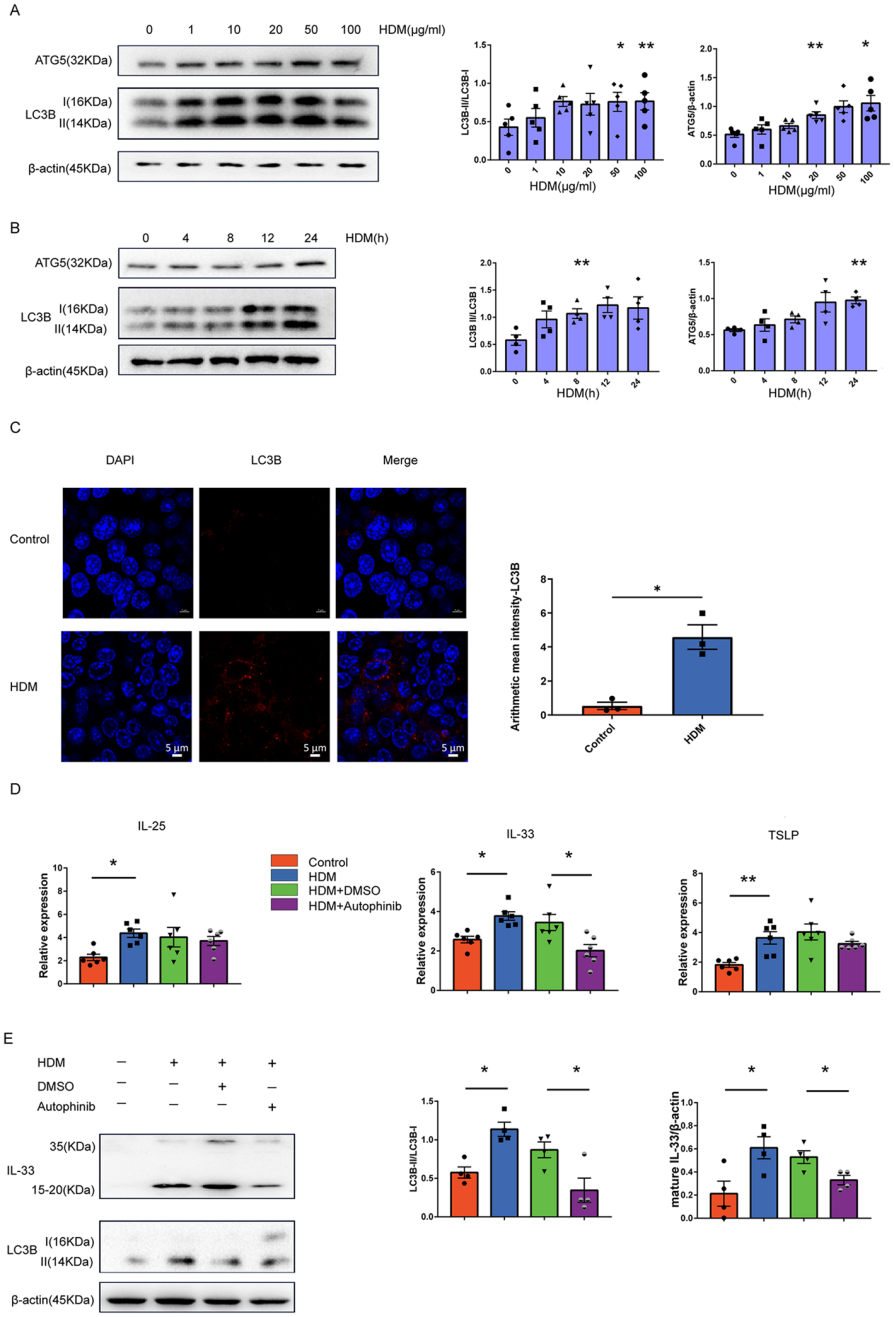
HDM mediated IL-33 production in epithelial cells by autophagy. **A** MLE-12 cells were left untreated or treated with different concentrations of HDM for 24 h, and the autophagy levels (LC3B and ATG5) were examined using a Western blot assay. The density quantification of LC3B and ATG5 was expressed as a ratio relative to β-actin. **B** MLE-12 cells were left untreated or treated with HDM extract (50 μg/mL) for the indicated time periods; then, autophagy levels were measured with Western blotting. The density quantification of LC3B and ATG5 was expressed as a ratio relative to β-actin (**C**) Representative immunofluorescence images showing increased autophagy levels after HDM treatment in MLE-12 cells. Scale bar, 5 μm. **D** MLE-12 cells were treated with HDM (50 μg/mL) alone or pretreated with autophinib. The expression levels of IL-25, TSLP, and IL-33 were measured with qPCR. **E** MLE-12 cells were treated with HDM (50 μg/mL) alone or pretreated with autophinib. The expression levels of autophagy and IL-33 were measured using a western blot assay and the density quantification of IL-33 was expressed as a ratio relative to β-actin. Data are presented as mean ± SEM of independent experiments with similar results (n = 3 ~ 6). *p < 0.05, **p < 0.01, ***p < 0.001, ****p < 0.0001

To date, we demonstrated that HDMs induced autophagy in AAI model and upregulated IL-33 in respiratory epithelial cells. We then explored whether autophagy regulated IL-33 expression. As shown in Fig. 4A, B LC3B-II/LC3B-I and ATG5 expression levels were significantly upregulated following HDM treatment. In addition, immunofluorescent staining indicated that HDM significantly increased the number of LC3B puncta, indicating autophagosome formation (Fig. 4C). To determine whether autophagy induction was involved in IL-33 production under HDM exposure, we treated MLE-12 cells with the autophagy inhibitor autophinib, followed by exposure to HDM. HDM-induced IL-33 production was partially blocked by autophinib pretreatment (Fig. 4D), but this was not the case for HDM-induced IL-25 and TSLP production. As shown in Fig. 4E, the efficiency of autophinib in inhibiting HDM-induced autophagy was verified by the reduction in LC3B-II/LC3B-I expression in autophinib-pretreated MLE-12 cells compared to cells treated with HDM plus solvent (DMSO). In addition, IL-33, especially in its mature form, was decreased in HDM-treated epithelial cells pre-exposed to autophinib. These findings together indicated that autophagy played a critical role in mediating IL-33 upregulation in HDM-treated epithelial cells.

### KIF2A decreased IL-33 expression and autophagy in epithelia cells

**Fig. 5:**
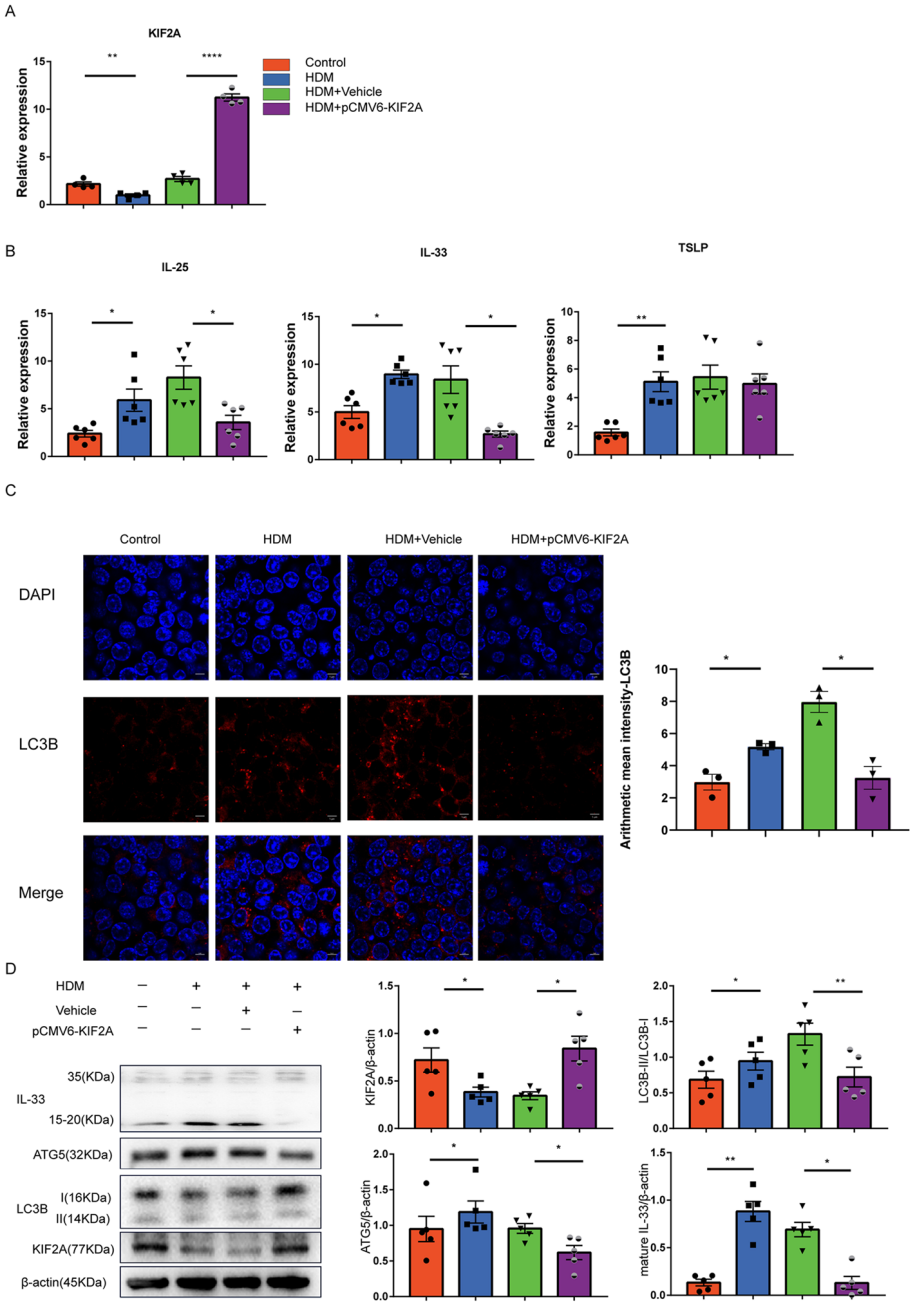
KIF2A mediated autophagy induction and IL-33 secretion in epithelial cells. **A** MLE-12 cells were transfected with pCMV6-KIF2A or vehicle and then exposed to HDM (50 μg/mL) 36 h after transfection. 24 hous after HDM exposure, the expression of KIF2A was examined using qPCR. **B** MLE-12 cells were left untreated or treated with HDM as described in (**A**). The TSLP, IL-25, and IL-33 expression levels were examined using qPCR. **C** Representative immunofluorescence images showing autophagy levels in different groups. Scale bar, 5 μm. **D** MLE-12 cells were transfected with pCMV6-KIF2A or vehicle and then exposed to HDM (50 μg/mL) 36 hous after transfection. The expression levels of KIF2A, autophagy, and IL-33 were examined 24 hous after HDM exposure with Western blotting and the density quantification was measured as a ratio relative to β-actin. Data are presented as mean ± SEM of independent experiments with similar results (n = 4 ~ 6). *p < 0.05, **p < 0.01, ***p < 0.001, ****p < 0.0001

After identifying the functional links between autophagy induction and IL-33 production in epithelial cells, we next explored whether KIF2A led to IL-33 production following HDM exposure via autophagy. Vehicle or pCMV6-KIF2A plasmids were separately transfected into MLE-12 cells. The transfection efficiency was verified by qPCR (Fig. 5A). Using qPCR, we detected the expression of IL-25, IL-33, and TSLP in MLE-12 cells overexpressing KIF2A in the HDM-induced response and found that IL-25 and IL-33 were downregulated (Fig. 5B). There was a significant reduction in the number of LC3B puncta in MLE-12 cells with KIF2A overexpression (Fig. 5C). We also observed that the upregulation of both LC3B and ATG5 upon HDM exposure was blocked by KIF2A overexpression. Similarly, IL-33 in HDM-treated epithelial cells was partially restored in KIF2A-overexpressing epithelial cells (Fig. 5D). Together, these results implied that KIF2A-autophagy pathway activation was essential for inducing IL-33 expression in HDM-treated epithelial cells.

### KIF2A regulated HDM-induced autophagy via mTORC1 signaling

**Fig. 6:**
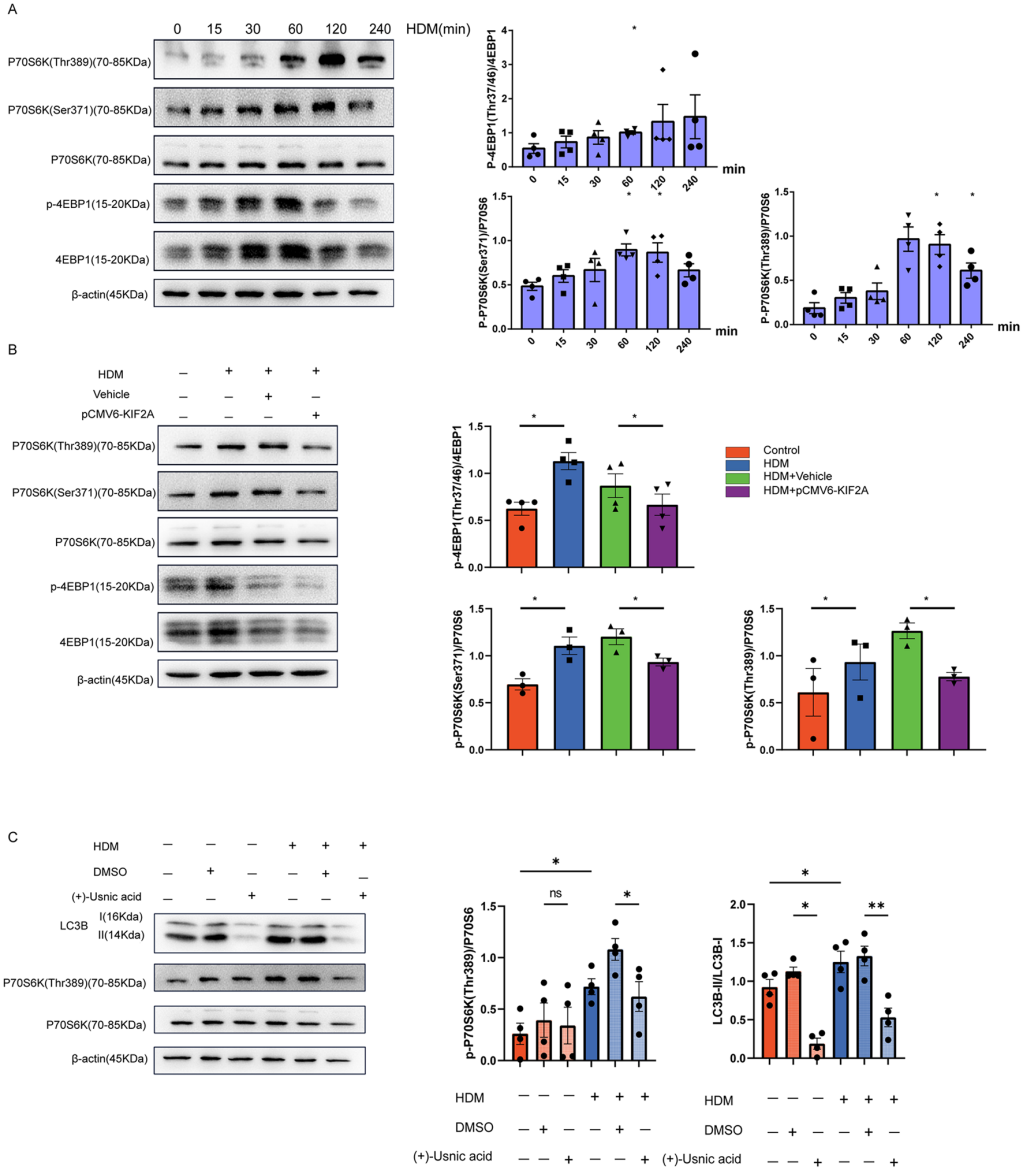
KIF2A regulated HDM-induced autophagy via mTORC1 signaling. **A** MLE-12 cells were left untreated or treated with HDM extract (50 μg/mL) for the indicated time periods; then, the activation status of mTORC1 was determined with Western blotting and quantification was based on band density. **B** MLE-12 cells were transfected with pCMV6-KIF2A or vehicle and then treated with HDM (50 μg/mL) 36 h after transfection. The activation status of mTORC1 was examined 2 h after HDM exposure with Western blotting and quantification was based on band density. **C** MLE-12 cells were treated with HDM (50 μg/mL) alone or pretreated with ( +) Usnicacid. The expression levels of autophagy and phosphorylation of P70S6K1 at Thr 389 were measured using a Western blot assay and and quantification was based on band density. Data are presented as mean ± SEM of independent experiments with similar results (n = 4). *p < 0.05, **p < 0.01, ***p < 0.001, ****p < 0.0001

Since we found that HDM decreased KIF2A expression in epithelial cells and KIF2A negatively regulated autophagy, we further determined the underlying mechanisms by which KIF2A mediated autophagy in HDM-treated epithelial cells. Some experiments with rapamycin support that heightened mTOR activity may be responsible for inhibition of autophagy [28]. mTOR regulates protein synthesis at synapses via two distinct downstream pathways, the p70 ribosomal S6 protein kinase (p70S6 kinase) and the eukaryotic initiation factor 4E (eIF4E) binding protein 4E-BP1, which are responsible for promoting translation of different pools of mRNAs [29]. We speculated that mTORC1 may regulate autophagy in HDM-treated epithelial cells. As evidenced by increased levels of P70S6K and 4EBP1 phosphorylation in HDM-treated-MLE-12 cells, (Fig. 6A), HDM exposure induced strong activation of P70S6K and 4EBP1 indicating that HDM provoked mTORC1 activity. In contrast, KIF2A overexpression blocked mTORC1 activation in MLE-12 cells in response to HDM stimulation (Fig. 6B). To determine whether mTORC1 was involved in autophagy positively under HDM exposure, we treated MLE-12 cells with the ( +)-Usnic acid to inhibit the the phosphorylation of S6K, followed by exposure to HDM. As shown in Fig. 6C, HDM-induced autophagy was blocked by ( +)-Usnic acid pretreatment. These data indicated that mTORC1 pathway activation might positively mediate the autophagy in HDM-treated MLE-12 cells.

### KIF2A attenuated airway inflammation

**Fig. 7:**
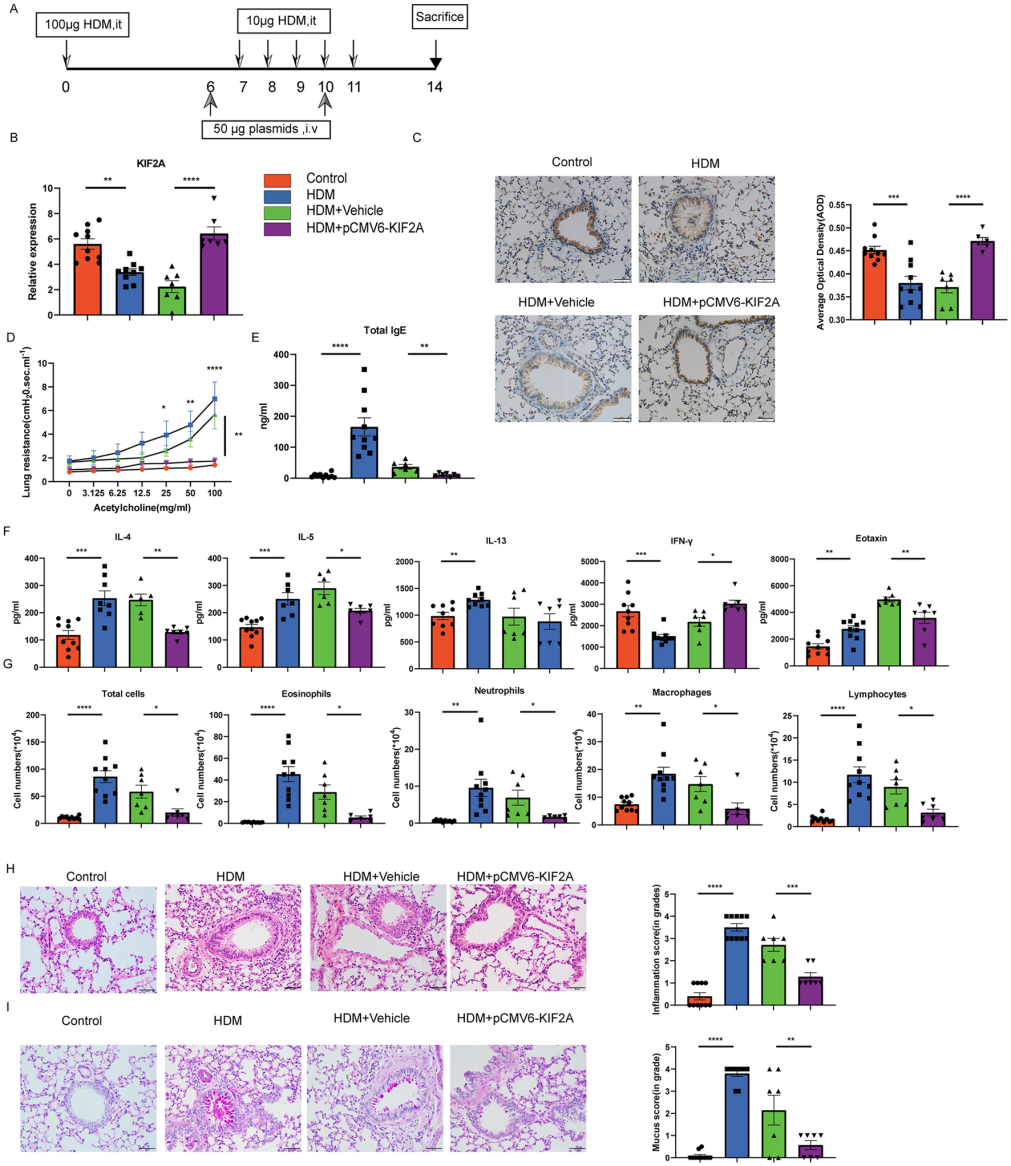
KIF2A attenuated allergic airway inflammation. **A** Experimental design of the study. **B** The level of KIF2A in different groups was examined using qPCR. **C** Immunohistochemical staining for KIF2A in lung sections from mice in different groups and AOD was measured with imageJ. **D** Mice inhaled increasing doses of acetylcholine (0–100 mg/mL), and AHR was measured. **E** The concentration of total IgE in seurm was measured with ELISA. **F** The concentrations of different cytokines (IL-4, IL-5, IL-13, Eotaxin and IFN-γ) in lung homogenates were measured with ELISA. **G** Total and differential cell counts in bronchoalveolar lavage fluid were determined by Wright-Giemsa staining. **H** Lung sections were stained with hematoxylin and eosin to analyze the infiltration of inflammatory cells, and the severity of peribronchial inflammation was graded semiquantitatively. **I** Lung sections were stained with PAS to assess goblet cell hyperplasia, PAS-positive and PAS-negative epithelial cells were counted, and the percentage of PAS-positive cells per bronchiole was calculated. Scale bar, 50 μm. Data are presented as mean ± SEM of independent experiments with similar results (n = 6 ~ 10). *p < 0.05, **p < 0.01, ***p < 0.001, ****p < 0.0001

To better understand the protective role of KIF2A in asthma pathogenesis, we overexpressed KIF2A in a murine model of AAI via intravenous administration of pCMV6-KIF2A using in vivo jetPEI (Fig. 7A). As shown in Fig. 7B, the expression of KIF2A was upregulated in the lungs of mice transfected with pCMV6-KIF2A compared to the lungs of mice transfected with vehicle, based on qPCR results. We further confirmed this observation using immunocytochemistry, as evidenced by the increased average optical density (Fig. 7C). Subsequently, KIF2A overexpression in allergic mice was associated with reduced AHR and total serum IgE (Fig. 7D, E). The increased IL-4, IL-5 and eotaxin levels and decreased IFN-γ levels in the lungs of mice treated with HDMs were significantly alleviated by KIF2A overexpression. However, IL-13 levels did not differ significantly between mice in the HDM + Vehicle group and those in the HDM + pCMV6-KIF2A group (Fig. 7F). Treatment with pCMV6-KIF2A induced decreases in the total cell counts and the number of eosinophils, neutrophils, macrophages, and lymphocytes (Fig. 7G). pCMV6-KIF2A-treated mice also exhibited diminished inflammatory infiltration around airway lumens and vessels compared to mice in the HDM + Vehicle group (Fig. 7H). Moreover, goblet cell hyperplasia and mucus secretion in the lumen of the bronchioles were significantly relieved (Fig. 7I). In summary, with the upregulation of KIF2A in the lung, type 2 pulmonary inflammation in a mouse model of allergic airway inflammation was significantly alleviated.

### KIF2A decreased IL-33 and autophagy in vivo

**Fig. 8:**
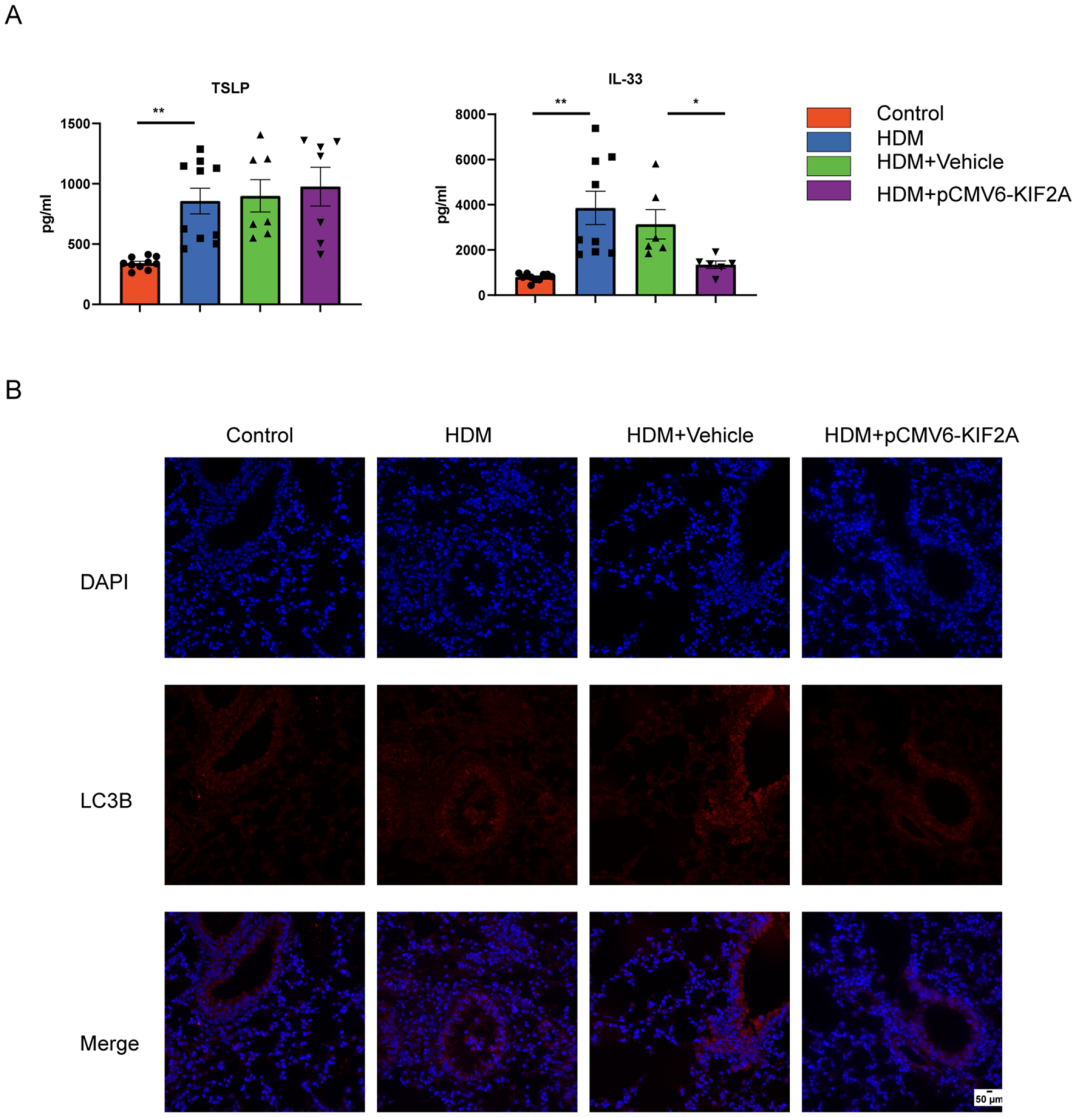
KIF2A decreased IL-33 and autophagy in vivo. **A** The concentrations of IL-33 and TSLP in lung homogenates were measured with ELISA. **B** Representative immunofluorescence images showing autophagy levels in different groups. Scale bar, 50 μm**.** Data are presented as mean ± SEM of independent experiments with similar results (n = 6–10). *p < 0.05, **p < 0.01, ***p < 0.001, ****p < 0.0001

We also detected TSLP and IL-33 levels in lung homogenate and found that IL-33 levels were decreased in HDM-treated mice via KIF2A overexpression, while TSLP levels did not differ significantly (Fig. 8A). We found increased immunofluorescent staining for LC3B in the airways of the mice exposed to HDMs compared to the airways of the control mice. However, pCMV6-KIF2A treatment relieved LC3B expression in the HDM-induced airway, as shown by immunofluorescence (Fig. 8B). These results suggested that KIF2A regulated the autophagic pathway and IL-33 expression, which may contribute to allergic airway inflammation.

## Discussion

RNA-seq showed that KIF2A was downregulated in differentiated asthmatic human airway epithelial cultures [16]. KIF2A in hypoxic dendridtic cells (DCs) may mediate Th2 polarization in vitro [30]. However, the functions of KIF2A in allergic asthma remain largely elusive. In this study, we first verified that KIF2A was decreased in a mouse model of allergic airway inflammation and that autophagy levels were upregulated. Furthermore, we found that KIF2A was downregulated while alarmin cytokines were upregulated in HDM-treated epithelial cells, which was attributed to autophagy. The KIF2A-mTORC1 axis was implicated in regulating autophagy and IL-33 production in the HDM-induced cellular response in vitro. In the HDM-induced allergic airway inflammation model, pCMV6-KIF2A treatment relieved airway hyperresponsiveness, total IgE, and pulmonary inflammation. Moreover, autophagy and IL-33 levels were decreased under pCMV6-KIF2A treatment. These results suggested that KIF2A protected epithelial cells for asthma initiation via suppressing mTORC1-autophagy/IL-33 pathway.

Autophagy has been implicated in asthma pathogenesis, i.e., double-membrane autophagosomes were more prevalent in epithelial cells from bronchial biopsy tissue of a moderately severe asthmatic patient than in the corresponding cells of a healthy subject [31]. The roles of autophagy in allergic asthma, however, are controversial. One study demonstrated that the autophagy stimulator simvastatin inhibited airway inflammation and airway remodeling through upregulation of autophagy in mouse models of asthma [32]. In contrast, another study showed that autophagy was activated in airway epithelium and airway smooth muscle, and the autophagy inhibitor chloroquine could significantly reduce airway inflammation, hyperresponsiveness, and structural remodeling in a mouse model of asthma [33]. Our results confirmed that autophagy levels were increased in the HDM-induced AAI model and pulmonary epithelial cells, which contributed to IL-33 production and disease severity. The complicated roles of autophagy in asthma warrant further research.

Lysosomes are dynamic intracellular organelles [34]. In autophagy, cytoplasmic proteins are engulfed by autophagosomes, which are transported along microtubules towards their minus ends and ultimately fuse with lysosomes [35]. KIF2A and the closely related kinesin KIF2C control lysosomal organization in human bronchial epithelial cells [36]. In addition, lysosomal positioning is thought to regulate autophagosome formation by influencing mTORC1 activity [17]. These previous studies guided us to study the relationship between KIF2A and autophagy in AAI. Currently, more than 40 kinesins have been found in mammals, and most of them have primary functions as cargo carriers. In contrast, kinesin family member (KIF) 2A depolymerizes microtubules rather than transports cargo [37]. Toll-like receptors (TLRs), upon HDM stimulation [38], may regulate the microtubule network by targeting kinesin family members [39, 40]. We postulated that HDM may decrease KIF2A via TLRs on epithelial cells.

Most studies have shown that IL-33 appears to downregulate the autophagic and inflammatory responses in the brain [41, 42]. Meanwhile, autophagy might regulate IL-33 secretion by modulating NF-κB in a model of acute lung injury [43]. Inflammatory serine proteases [8, 44] and allergen proteases [9] could cleave the IL-33_FL_ precursor into mature forms of IL-33. The data from the present study revealed that autophagy regulated the maturation of IL-33 under HDM treatment in epithelial cells and in a mouse model of AAI. However, the mechanisms by which autophagy regulate IL-33 maturation remains to be explored.

Canonical mTORC1-autophagy pathway indicates mTORC1 is a negative regulator of autophagy [45, 46]. However, involvement of P70S6K1, an mTORC1 substrate, in autophagy regulation is controversial. p70S6K1 negatively regulates autophagy in rat hepatocytes [47]. Positive regulator of autophagy Atg1 (an ortholog of the mammalian ULK1), has been shown to inhibit P70S6K1 activity in Drosophila and mammalian cells by blocking phosphorylation of P70S6K1 at Thr 389 [48]. Other data has evidenced that inhibition of S6K1 phosphorylation induces autophagy which leads to death of Drosophila and some mammalian cell lines [49]. In fact, p70S6K1 is necessary for autophagy in the fat body of Drosophila melanogaster [50, 51]. Moreover, inhibition of S6K1 by resveratrol attenuated autophagy induced by nutrient limitation or rapamycin in a number of mammalian cell lines [52]. In addition, endothelin-1 stimulates phosphorylation of mTORC1 targets (p70S6K, 4EBP1, Ulk1) and increases LC3-II and p62–consistent with mTORC1 activation and enhanced autophagy in myocytes [53]. Recent study demonstrated that allergen-initiated inflammation suppresses mTOR and induces autophagy in airway epithelial cells, resulting in the production of certain proallergic cytokines such as IL25, further promoting the type 2 response and eventually perpetuating airway inflammation in asthma [54]. In contrast, we demonstrated that mTORC1 pathway activation positively mediated autophagy in HDM-treated epithelial cells (MLE-12), which further suggested the effects of mTORC1 in autophagy may rely on cell type and stimulus conditions.

## Conclusion

In conclusion, HDM specifically down-regulated KIF2A, which led to increased levels of autophagy and IL-33 in epithelial cells via the activation of mTORC1 signaling pathway. In a mouse model of AAI, KIF2A over-expression mitigated disease severity, IL-33 production, and autophagy. Our research mainly depends on the traditional cell culture and mouse model of asthma. The differentiated primary human airway epithelial cells using air–liquid interface culture and clinical samples should be tested in the future. Nevertheless, our findings suggest that KIF2A might be a promising therapeutic target in allergic diseases.

## Supplementary Information

**Additional file 1: Figure S1. **KIF2A was downregulated in asthmatic airway epithelial cells. The expression value of KIF2A in asthma and non-asthma group according to the reference (*PloS one* 2015; https://doi.org/10.1371/journal.pone.0118286). Data were presented as mean ± SEM

## Data Availability

All data that support the findings in this study are available from the corresponding author upon reasonable request.
