# Supplementary figures and images for: KIF2A decreases IL-33 production and attenuates allergic asthmatic inflammation
Source: Allergy Asthma Clin Immunol. 2022 Jun 19;18:55. doi: 10.1186/s13223-022-00697-9 (PMC9208156; doi:10.1186/s13223-022-00697-9)

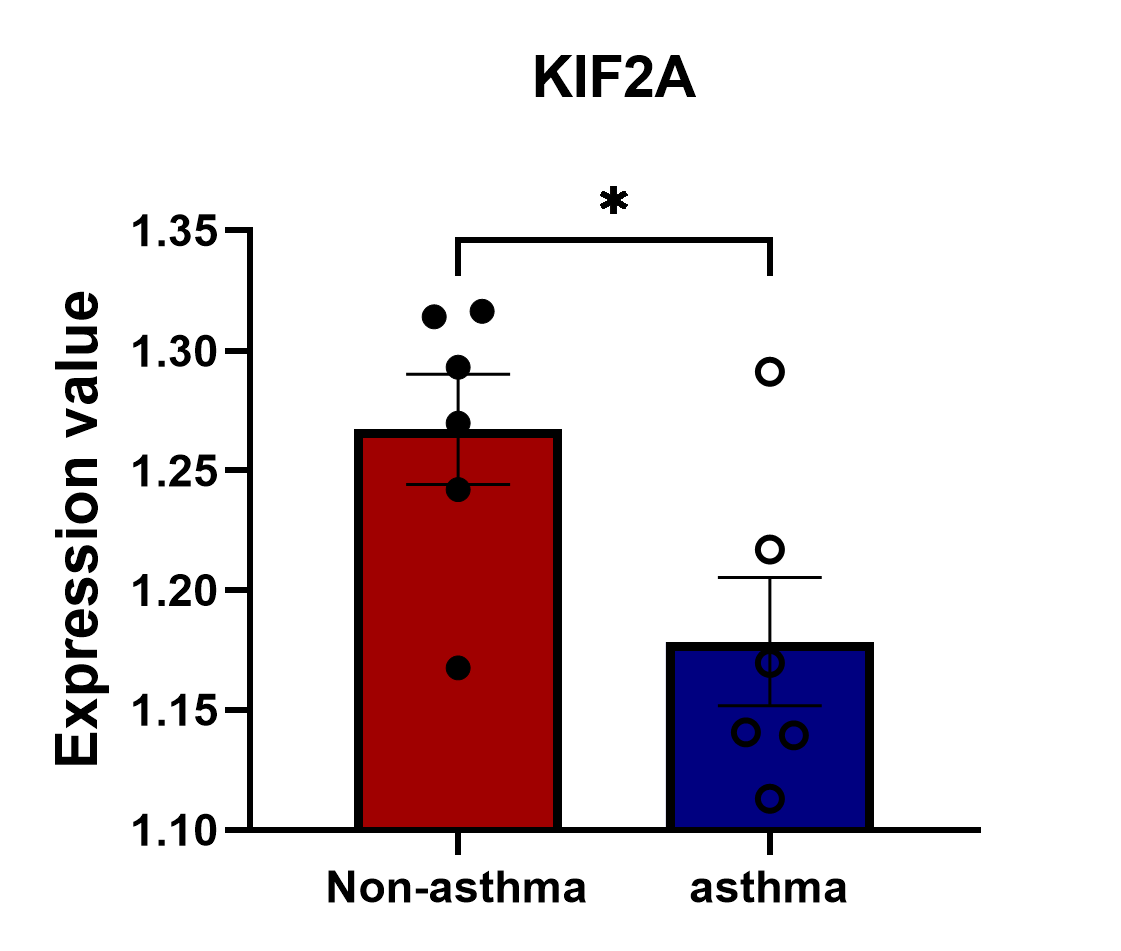

Supplement: Supplementary file 1 — Additional file 1: Figure S1. KIF2A was downregulated in asthmatic airway epithelial cells. The expression value of KIF2A in asthma and non-asthma group according to the reference (PloS one 2015; https://doi.org/10.1371/journal.pone.0118286). Data were presented as mean ± SEM [file 13223_2022_697_MOESM1_ESM.tif]
